# Transfer Rates of ^238^U and ^232^Th for *E. globulus, A. mearnsii, H. filipendula* and Hazardous Effects of the Usage of Medicinal Plants From Around Gold Mine Dump Environs

**DOI:** 10.3390/ijerph121215021

**Published:** 2015-12-10

**Authors:** Victor M. Tshivhase, Raymond L. Njinga, Manny Mathuthu, Thulani C. Dlamini

**Affiliations:** Centre for Applied Radiation Science and Technology, North West University, Mafikeng 2735, South Africa; Victor.Tshivahase@nwu.ac.za (V.M.T.), Manny.Mathuthu@nwu.ac.za (M.M.); thukscd@gmail.com (T.C.D.)

**Keywords:** transfer factor, uranium, thorium, tailings storage site, contaminated soil

## Abstract

Medicinal plant consumption can be a source of human exposure to radioactive elements such as ^238^U and ^232^Th, which can lead to internal radiation doses. The uptake of ^238^U and ^232^Th from soils to the leaf samples of three different medicinal plant species (*Eucalyptus globulus, Acacia mearnsii and Hyparrhenia filipendula*) from the purlieu of the Princess gold mine dump, an abandoned contaminated tailings storage site (TSS), located at longitude 27°55′00″E and latitude 26°09′30″S in Davidsonville (Roodepoort, west of Johannesburg, South Africa) was measured. This was done using ICP-MS spectrometry and substantial differences were observed in the soil-plant transfer factor (TF) values between these radionuclides. The plant species *E. globulus* exhibited the highest uptake of ^238^U, with an average TF of 3.97, while that of *H. filipendula* was 0.01 and the lowest TF of 0.15 × 10^−2^ was measured for *A. mearnsii*. However, in the case of ^232^Th, the highest average TF was observed for *A. mearnsii* (0.29), followed by *E. globulus* (0.10) and lowest was measured for *H. filipendula* (0.27 × 10^−2^). The ratio of TF average value *i.e.*, ^238^U to ^232^Th in the soil-plant leaves was 38.05 for *E. globulus*, 0.01 for *A. mearnsii* and 4.38 for *H. filipendula*.

## 1. Introduction

In Africa, the therapeutic use of plants in traditional health practices is common and widespread, predating the introduction of antibiotics and other modern drugs [1,2]. More than 80% of the World’s population still depends on traditional medicines for various diseases [3,4], and natural products have been used worldwide for medicinal purposes for thousands of years, [5]. Olajuyigbe and Afolayan [6] studied the medicinal potential of *Acacia mearnsii* and revealed that the plant is medicinal. The leaves of this plant are widely used by traditional healers in South Africa in the treatment of microbial infections. *A. mearnsii*, commonly known as Black Wattle tree, grows to about 15 m high and is a short medium-lived woody perennial and spreading tree. The plant is smooth and has a greenish-brown bark on the young branches which are blackish and rough on the trunk. The young branchlets are silky and the plant is widespread in lowlands, open forest, cleared land, heathy woodland, and particularly on dry, shallow soils [7,8]. The plant has globular inflorescences with 20–30 tiny pale yellow flowers. The color of the pods are dark brown to black, constricted between the seeds, and are more or less straight having a wide of 5–8 mm and are about 5–10 cm long [7]. 

Eucalyptus oil, which comes from the dried leaves of the *E. globulus*, is colorless with a strong woody and sweet scent [9]. The oil has multiple different uses. It is often a key ingredient in perfumes and cosmetics because of its unique fresh and clean aroma, and is also used as a dental or industrial solvent. The plant is used in China and India for a range of medical conditions. It has antibacterial effects on pathogenic bacteria of the upper respiratory tract. Trivedi and Hotchandani [9] published that extract from the leaves can treat bladder diseases, fever, diabetes, flu, ulcers, acne, wounds and burns, arthritis and stuffed nose. 

*H. filipendula* is use for domestic grazers and as fodder plant for wild animals. When young, the grass provides fair to good grazing and food; at later stages it becomes too coarse to be suitable as forage, although it is still considered edible by humans and livestock during the dry season in Sudan [10]. It is also used for thatching and weaving in South Africa and other countries like Nigeria and Cameroon. It is widely distributed in East and southern Africa, from Sudan, Ethiopia and Kenya south- and westward to Angola and South Africa. In traditional medicine a decoction of the root is taken in Zimbabwe against syphilis [10,11].

The consumption of medicinal plants can lead to internal radiation doses and thus act as a source of human exposure to radioactive elements [12,13]. The radioactive waste (e.g., tailings) produced by mining activities may contain a series of long-lived radionuclides, such as uranium (U), radium (Ra), and thorium (Th) isotopes. It has been estimated [14] that the annual effective dose of long-lived natural radionuclides (^232^Th, ^238^U, ^21^°Pb, ^226^Ra and ^228^Ra), due to the ingestion of leafy vegetables, fruits and roots of some plants and their derived products by adult inhabitants of Rio de Janeiro City (Brazil) reached 14.5 mSv [15,16,17,18,19]. 

Africa has a large share of the World’s mineral resources and biodiversity, and consequently also of the responsibilities and liabilities associated with mineral extraction. Mineral and mining companies in South Africa are not always responsible for environmental management. The challenges of managing the environmental footprints of mining regions and abandoned mines remaining in the country are now being addressed [20]. Roodepoort has developed into a densely populated and industrialised region of South Africa since gold mining began in the late 1800s. Estimates of the area utilised for mine residue deposits and gold tailings storage sites (TSS) range from 400 to 500 km^2^ of the 1600 km^2^ in Witwatersrand. Many residues and TSS are situated in densely populated areas, and the zone of influence is extensive [21]. In addition to dust generation, the TSS are sources of acid rock drainage, resulting in the movement of low pH, saline water and leached metals, including naturally occurring radionuclides (NORs), into soils, groundwater and entire watersheds [21,22,23,24,25]. 

People living closer to these TSS tend to be worse-affected by environmental dynamics since they may depend on some of these plants for building thatch houses, roofing, animal grazing, traditional medicines, weaving, and household implements, among other uses [26,27,28,29]. Njinga *et al.*, [30], revealed that elevated concentrations of NORs in plants could pose a health risk to humans or animals if sufficient quantities are ingested in the form of herbal medicine. A few medicinal plants and traditional medicines have been found to contain increased concentrations of NORs, although the doses administered are usually low and unlikely to pose a health risk [30,31,32,33,34,35]. For some decades, few work on radioactive food contamination in the environment and its pathway mechanism to plants, animals and human has been reported [12,36]. Little record of radioactive contamination of the environment has been reported around the Princess Mine TSS, Roodepoort, South Africa. 

Migration of radionuclides in the soil-plant system is complex and the transfer factor (TF) assessment models is commonly utilize to estimate the pathway of radionuclides through the food chain [37]. This ratio describes the concentration of radionuclide expected to be in a plant based on the concentration in the soil. According to Bettencourt *et al.* [38], the physico-chemical form of the radionuclides, type of plants and the part of the plant in concerned influences the TF. 

The uptake of long-lived radionuclides among different plant species are not the same. Over the past decades, several investigations on mobilization of natural radionuclides (^238^U and ^226^Ra) in different compartments (soil, plant, and water), as well as the transfer between them, have been performed at different mining sites around the world [17,39,40].

This study aimed to investigate the soil-to-plant transfer factors of ^238^U and ^232^Th and the uptake in the abandoned Princess gold mining TSS since there are no TF data for these plant species. A slightly elevated concentration of these radionuclides has been detected in some of the soils and plants. 

### Geology of the Study Site

**Figure 1:**
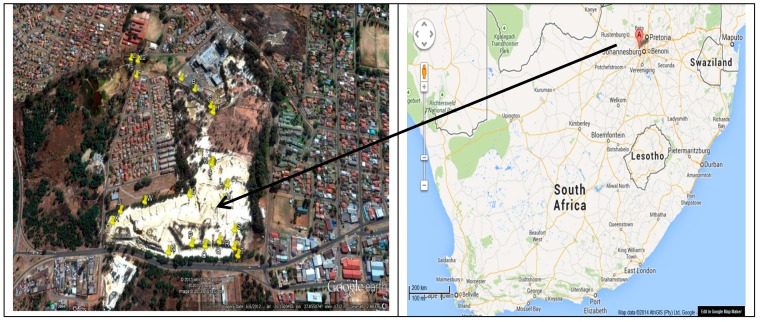
Sampling TSS marked “yellow” (**left**) of the study area marked “A” (**right**).

The Princess Dump site is an old and abandoned gold tailings dam located in Davidsonville, Roodepoort, located west of Johannesburg at longitude 27^o^55′00″E and latitude 26^o^09′30″S, approximately 3 km north-west of the Durban Roodepoort Deep Gold Mine (DRD). The general location of the study area is marked as point A in Figure 1. The top of the mine dump is generally devoid of any vegetation, but there are plenty of trees and grass around it.

The original site of Princess Gold Mine including the TSS comprises a thick sequence of sediments overlain by Achaean sedimentary rocks. The surface geology of the site is dominated by quartzite and conglomerates of the Central Rand Group, a sub division of the Witwatersrand Super group. Fresh, hard rock dolomite is encountered at depths ranging from 0 to 50 m below ground [41]. The soils are predominantly sandy loams, red, yellowish with low to medium base status [42] derived from the weathering of the underlying dolomite and quartzite. 

The TSS site is highly susceptible to erosion which in turn results in a high sediment load that end up in water streams increasing the water course siltation, water turbidity and decreasing the water quality around the site [41]. The TSS area (Figure 1) falls within the Upper Klip River sub-catchments, a tributary to the Vaal River. The exact date of deposition of the tailings is unknown, but records indicate that the gold tailings dam predates 1933 [42]. Much of the property on the site falls within the City of Johannesburg, with a smaller portion owned by Grifo Property Investments [42]. The site has not been closed or rehabilitated to protect the environment and human health from pollution risks.

## 2. Experimental and Analytical Method

### 2.1. Sample Analysis Using the NexION 300Q ICP-MS Instrument and Technique

Measurement using ICP-MS was performed at the North West Universality, Centre for Applied Radiation Science and Technology. The NexION 300Q ICP-MS instrument (Perkin Elmer, Waltham, MA, U.S.A.) used in this study for analyzing radionuclides has been accredited according to ISO standard17025 (European Standard EN ISO/IEC17025:2000). A solution analytical method was used with internal multi-standard calibration method [43].

### 2.2. Sample Preparation for ICP-MS 

Around each medicinal plant, an area of 5 m^2^ was designated and four soil samples were collected at the edges while two were collected along the diagonal mid-point closest to the plant linking the four marked edges. Therefore, around a selected plant species, six different soil samples of 1 kg each were collected at 1 m depths each using an auger of 10 cm diameter and thoroughly mixed to form a 1 kg composite soil sample. This was done after the vertical or near vertical surface was dressed (scraped) to remove smeared soil to minimize the effects of contaminant migration interferences due to smearing of material from other levels. For *E. globulus* the plants were labelled “L-Glob to G-Glob” while the composites soil samples labelled “L-Glob-SL to G-Glob-SL”. For *A. mearnsii*, the plants were labelled “L-MEARN to O-MEARN” and the composites soil samples labelled “L-MEARN-SL to O-MEARN-SL”. For *H. filipendula* the plants were labelled “J-FILI and S-FILI” and the composite soil samples labelled “J-FILI and S-FILI”. The plant leaves from each species were harvested and mixed thoroughly then packaged in perforated bags. The above samples were transported to the laboratory for analysis.

1 kg of each soil sample was air-dried and passed through a 2-mm mesh sieve. The medicinal leaves were washed with deionized water at least three times to remove dust and soil particles. After air-drying for 14 days they were ground using an electric mill. The leaves were ashed for 18 h at 600 °C. For the measurement of the nuclides, samples were prepared as follows, the soils and medicinal leaf samples, 100 mg each, were digested with mineral acids (a mixture of 9 mL diluted HCl and 3 mL diluted HNO_3_) using a MARS 5 microwave digester (CEM GmbH, Kamp-Lintfortstate, Germany). Following their digestion, both the soil and medicinal plant leaf samples were evaporated to near dryness at 800 °C for another 18 h and the dry ash weight of the plant leaves recorded. Finally, the residue was dissolved with 1 mL of 40% HNO_3_ and diluted with deionized water. The digested samples (*aqua regia* method) were filtered through the Whiteman number 42, diluted into 100 mL of deionized water. All the acids used were ultra-pure analytical grade (AA-100, Tama Chemicals Co, Kanagawa, Japan) Water (>18:1 MΩ) which was treated by a Milli-Q water system (Millipore Co., Massachusetts, MA, USA) was used throughout the work. After diluting the acid solutions to a suitable concentration, radionuclides in both the medicinal plant leaves and soil samples were measured using ICP mass spectrometry. For accurate determination of elemental compositions in the samples, a Perkin Elmer Pure Plus NexION Dual Detector Calibration Solution as the Atomic Spectrometric Standard, with the specifications: 200 micro-grams per liter of Al, Ba, Ce, Co, Cu, In, Li, Mg, Mn, Ni, Pb, Tb, U and Zn were used. 

## 3. Result and Discussion

The elemental concentrations in terms of mg·kg^−1^ of uranium or thorium were converted into activity concentration of ^238^U and ^232^Th [44,45]. From the result obtained from the ICP-MS, the average values for uranium and thorium concentration in the soil samples around *E. globulus* were 1.79 ± 0.32 and 0.82 ± 0.13 mg·kg^−1^ respectively. For the leaves of *A. mearnsii,* the average values were (1.3 ± 0.18) × 10^−3^ and (75.5 ± 12.52) × 10^−3^ mg·kg^−1^ while for the soil sample around the plant, the mean values were 3.66 ± 2.12 and 2.54 ± 1.13 mg·kg^−1^ for uranium and thorium respectively. Here, the concentrations were relatively higher in the soils compared to the leaves. Also for the leaves of *H. filipendula*, we obtained the mean value of (12.5 ± 5.12) × 10^−3^ and (1.73 ± 0.72) × 10^−3^ mg·kg^−1^ which were very low while the composite soil around the two locations for *H. filipendula*, the average values were 1.00 ± 0.14 and 0.85 ± 0.43 mg·kg^−1^ for uranium and thorium respectively. It was observed that uranium concentration was highest on the average in the soil around *A. mearnsii* with a value of 3.66 ± 2.12 mg·kg^−1^. 

### 3.1. Soil-to-Plant Transfer Factors (TF) 

TF value is defined as the concentration of a radionuclide in a plant (in Bq·kg^−1^ dry weight) divided by the concentration of the radionuclide in the soils (in Bq·kg^−1^ dry weight). This factor (TF) is the uptake of radionuclides by plants from the soils and were calculated using the element concentration data in the soils and the medicinal plant leaf samples. Equation 1.0 was used to obtain the specific activity concentration “in Bq·kg^−1^” for ^232^Th and ^238^U and the TF were evaluated as an index for the radionuclides accumulation by the plant leaves or the transfer of radionuclides from soil to plant leaves [37,46].

#### 3.1.1. Analysis of ^238^U Soil-to-Plant TF

**Figure 2:**
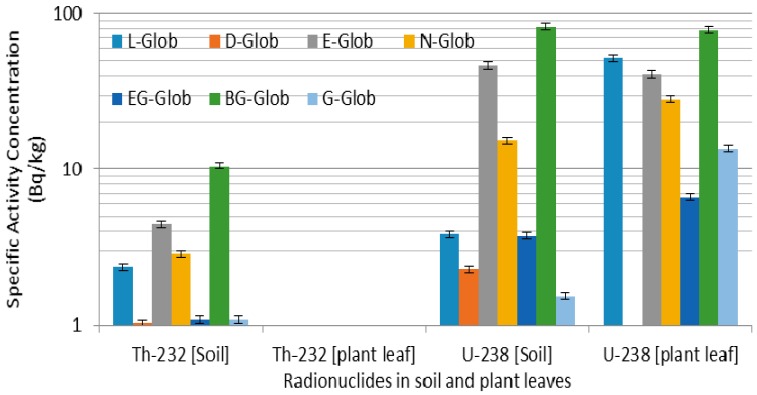
Specific activity distribution of ^232^Th and ^238^U for *E. globulus.*

**Figure 3:**
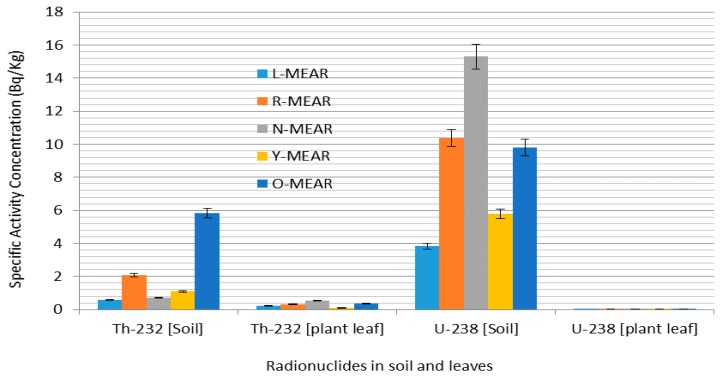
Specific activity distribution of ^232^Th and ^238^U for *A. mearnsii.*

**Figure 4:**
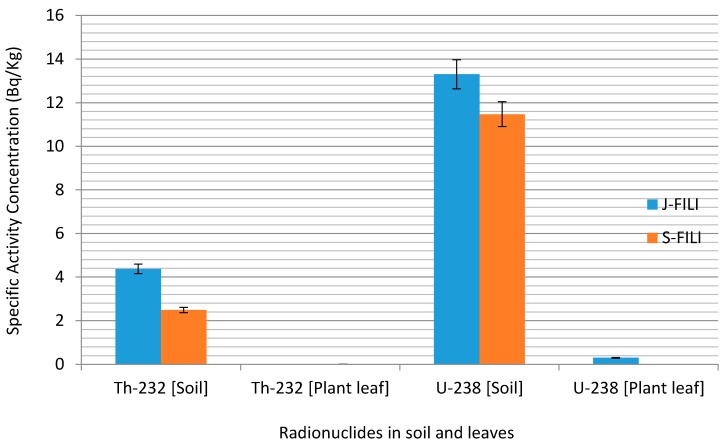
Specific activity distribution of ^232^Th and ^238^U for *H. filipendula.*

The observed mean concentration of specific activity of ^238^U (Bq·kg^−1^ dry weight) and the TF for ^238^U of the soil (L-Glob-SL to G-Glob-SL), (L-MEAR-SL to O-MEAR-SL) and (J-FILI-SL to S-FILI-SL) for the three plant species (*E. globulus*), (*A. mearnsii*), and (*H. filipendula*) are shown in Figure 2, Figure 3 and Figure 4 respectively. From Figure 2, it is observed that the specific activity of ^238^U for *E. globulus* was higher (22.22 ± 4.69 Bq·kg^−1^) compared to *A. mearnsii* (9.02 ± 3.41 Bq·kg^−1^) and *H. filipendula* (12.39 ± 3.52 Bq·kg^−1^) in the soil samples. However, in a similar trend, the mean specific activities of ^238^U were 31.36 ± 9.40 Bq·kg^−1^, 0.02 ± 0.01 Bq·kg^−1^ and 0.16 ± 0.14 Bq·kg^−1^ for the leaf samples of *E. globulus, A. mearnsii,* and *H. filipendula*, respectively. The average activity concentration of ^238^U in the leaves of *E. globulus* is of one and half order of magnitude greater relative to the soils around the TSS and the extract from the leaves are used by herbs dealers as therapy for bladder diseases, fever, diabetes, flu, ulcers, acne, wounds and burns, arthritis and stuffed nose. 

In the case of ^232^Th the average activity concentration in soil was 3.35 ± 1.64 Bq·kg^−1^ compared to 0.19 ± 0.06 Bq·kg^−1^ in the leaves of *E. globulus* (Figure 2). The same trend of ^232^Th were observed for *A. mearnsii* with average values of 2.06 ± 0.89 Bq·kg^−1^ in soil and 0.3066 in the leaves and *H. filipendula* with average values of 3.43 ± 0.27 Bq·kg^−1^ in soil and 0.11 ± 0.01 Bq·kg^−1^ in the leaves as seen in Figure 3 and Figure 4.

However, the TF for different plants are consistently larger in leaves of the *E. globulus* with lower concentration of ^238^U in the soils (average value of 3.97). However, a high TF of 13.44 was recorded for ^238^U in *E. globulus* for the L-Glob location. This means that much accumulation of the ^238^U was found in the leaves of *E. globulus* compared to the soil around that point which was identified to be very close to the TSS. This high value may also be due to other anthropogenic activities around the TSS. These industrialized activities might release radioactive particles that will bind to dust and aerosol particles and at later stage, deposited on the leaves of the vegetation and will be absorbed [47]. The average TFs of ^238^U are 3.97, 0.15 × 10^−2^ and 0.01 for *E. globulus, A. mearnsii and H. filipendula* respectively. This was generally in agreement with reported values for plants in contaminated soils [47,48]. However, for these plants growing in this locations, the variation of the ^238^U TF was expected because of the characteristics of the different plants. Nevertheless, relatively large variations were found between these plants. Among the plant species, the highest TF of 13.44 and 8.89 for tailings storage sites (TSS) contaminated soils (L-Glob-SL and G-Glob-SL respectively) for ^238^U were found for *E. globulus*. 

The high TF imply more radionuclides in the leaves and this species (*E. globulus*) is used for a range of medical conditions in traditional Chinese and Indian medicine and in South Africa. It has antibacterial effects on pathogenic bacteria in the upper respiratory tract. In the case of *A. mearnsii* which are used in the treatment of microbial infections in South Africa by traditional healers, the average TF was 0.15 × 10^−2^ and was very low compared to *E. globulus* In contrast, *H. filipendula* (used for wild and domestic grazers and eaten during the dry season in Sudan while in South Africa they are used for thatching and weaving and in Zimbabwe a decoction of the root is taken against syphilis) exhibited the low average TF of 0.01 on the soils (J-FILI-SL and S-FILI-SL). Among these three plant species with their natural metabolic differences, the difference in mean ^238^U TF were found to vary by a factor of about 5.27.

#### 3.1.2. Analysis of ^232^Th Soil-to-Plant TF

**Figure 5:**
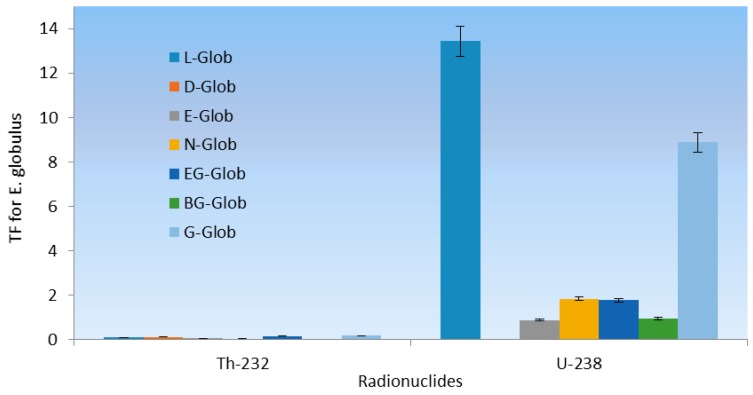
Soil to plant TF for ^232^Th and ^238^U around *E. globulus.*

**Figure 6:**
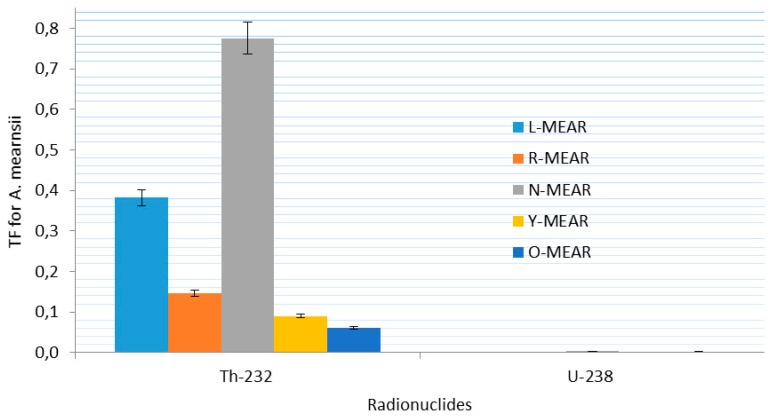
Soil to plant TF for ^232^Th and ^238^U around *A. mearnsii.*

**Figure 7:**
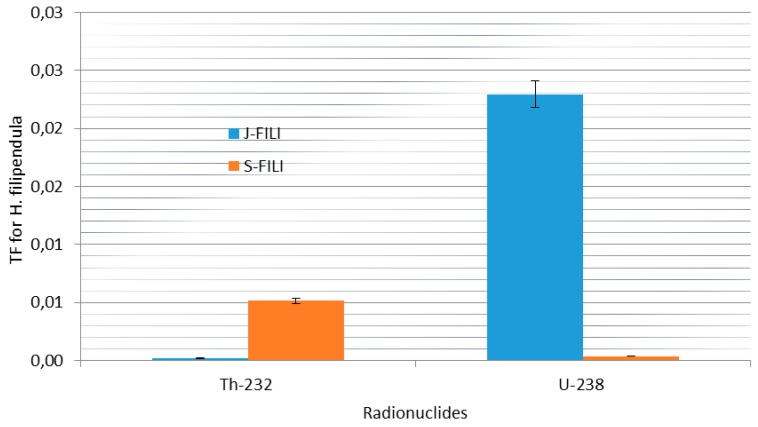
Soil to plant TF for ^232^Th and ^238^U around *H. filipendula.*

Figure 5, Figure 6 and Figure 7 present the corresponding specific activities and geometric mean TF of ^232^Th observed in this study across all plants. The mean specific activities of ^232^Th were 3.34 ± 1.61 and 0.19 ± 0.03 Bq·kg^−1^ for the soil sample types and the leaves of *E. globulus*, respectively. In contrast to ^238^U, the leaves mean TF of ^232^Th, 0.10 for *E. globulus*, 0.29 for *A. mearnsii* and 0.27 × 10^−2^ for *H. filipendula* were obtained and on the average was 10.01 order-of-magnitude lower than that of ^238^U. As shown in Figure 5, Figure 6 and Figure 7, the leaves uptakes of ^232^Th by *E. globulus* are at least 0.36 and 38.66 times greater than the leaves uptake of *A. mearnsii* and the leaves uptake of *H. filipendula* respectively in the soils around the TSS. As a result of numerous physical, biological, and chemical conditions in the soil and plant species, the uptake of the radionuclides will be affected. These gross effects and the individual chemical properties of the radionuclides, tend to affect the ^238^U and ^232^Th uptake by the plants. This concept was supported by [49] where it was reported that corn kernels exhibited low accumulation of Pu isotopes compared to other parts of the corn plant. Also, it was revealed [50] that legumes stored more radioactivity than grasses. The TF for ^232^Th were also consistently higher in S-FILI, N-MEARN and L-Glob species (Figure 5, Figure 6 and Figure 7). This shows that, ^232^Th TF for plant increased with decreasing activity concentration in the soils [19,40]. 

The rankings of transfer factors (TF) by different plant leaves for each radionuclides were as follows: G-Glob > EG-Glob > D-Glob > L-Glob > E-Glob > N-Glob > BG-Glob (*E. globulu*), N-MEAR > L-MEAR > R-MEAR > Y-MEAR > O-MEAR (*A*. mearnsii) and S-FILI > J-FILI (*H. filipendula*) for ^232^Th while L-Glob > G-Glob > N-Glob > EG-Glob > BG-Glob > E-Glob > D-Glob (*E*. globulu), N-MEAR > O-MEAR > Y-MEAR > R-MEAR > L-MEAR (*A. mearnsii*) and J-FILI > S-FILI (*H*. *filipendula*) for ^238^U. Our observed ranges of average TF for ^232^Th tended to be about 5 order-of-magnitude lower than that for ^238^U. In all cases, *E. globulus*, on the average, exhibited relative higher uptake for ^232^Th and ^238^U than other plant leaf species. As for ^238^U, J-FILI was high for the TF of *H. filipendula*, while relatively low for *A. mearnsii* compared to ^232^Th (Figure 6). However, *E. globulus* had significantly higher ^238^U activity concentrations than other plants. 

The ratio of TF average values *i.e.*, ^238^U to ^232^Th in the soil – plant leafs was 38.05 for *E. globulus*, 0.01 for *A*. *mearnsii* and 4.38 for *H. filipendulla* respectively. In general, the comparative uptake of ^238^U and ^232^Th by different plants is affected by numerous physical, chemical and biological conditions of the soil. These effects and the individual chemical properties of the nuclides, tend to affect its uptake by plants. For example, retention of radionuclides onto the soil particles will affect their availability for plant uptake. Martınez-Aguirre *et al.* [51] reported that thorium exhibited a much lower mobility than uranium, which is consistent with our observations that ^232^Th has smaller TF. The magnitude and range of TF of ^238^U and ^232^Th found in this study appeared to be generally similar to values obtained in other studies where radionuclides uptake was the primary point of focused [47,52,53]. However, the overall transfer factor values, obtained for a given radionuclide from the corresponding activity in a plant leaves did not immediately yield quantitative information on the translocation of this radionuclide from soil to other portion of the plants such as root, shoot tree backs, and branches which are commonly used for herbal therapy. However, since these species are directly involved in the human food chain whether in the form of herbal intake, wild and domestic cattle grazing or eaten directly, information on the concentration level and transfer of radionuclides from tailings will provide important data for the environmental risk assessment at such sites. 

## 5. Conclusions 

The TF, used as a parameter for the accumulation of radionuclides by plant leaves or the transfer of radionuclides from soil to plant leaves has been evaluated for three medicinal plant species (*Eucalyptus globulus, Acacia mearnsii and Hyparrhenia filipendula*). The TF of ^238^U and ^232^Th for three medicinal plant species on soil contaminated with gold mine tailings site in Roodepoort (South Africa) indicated that *E. globulus* had higher TF for both ^238^U and ^232^Th. Extracts from the leaves of *E. globulus* are of multi-traditional importance in the therapy of illness like bladder diseases, fever, diabetes, flu, ulcers, acne, wounds and burns, arthritis and stuffed nose. In general, the findings obtained from this study show that the leaves of *E. globulus*, *A mearnsii and H. filipendula* have high TF of the radionuclides studied. 
